# Estimating Incidence Curves of Several Infections Using Symptom Surveillance Data

**DOI:** 10.1371/journal.pone.0023380

**Published:** 2011-08-24

**Authors:** Edward Goldstein, Benjamin J. Cowling, Allison E. Aiello, Saki Takahashi, Gary King, Ying Lu, Marc Lipsitch

**Affiliations:** 1 Center for Communicable Disease Dynamics, Department of Epidemiology, Harvard School of Public Health, Boston, Massachusetts, United States of America; 2 The University of Hong Kong, Hong Kong Special Administrative Region, Hong Kong, China; 3 Center for Social Epidemiology and Population Health, University of Michigan, Ann Arbor, Michigan, United States of America; 4 Department of Applied Mathematics, Harvard University, Cambridge, Massachusetts, United States of America; 5 Institute for Quantitative Social Science, Harvard University, Cambridge, Massachusetts, United States of America; 6 Department of Humanities and Social Sciences in the Professions, Steinhardt School of Culture, Education and Human Development, New York University, New York City, New York, United States of America; 7 Department of Immunology and Infectious Diseases, Harvard School of Public Health, Boston, Massachusetts, United States of America; Albert Einstein College of Medicine, United States of America

## Abstract

We introduce a method for estimating incidence curves of several co-circulating infectious pathogens, where each infection has its own probabilities of particular symptom profiles. Our deconvolution method utilizes weekly surveillance data on symptoms from a defined population as well as additional data on symptoms from a sample of virologically confirmed infectious episodes. We illustrate this method by numerical simulations and by using data from a survey conducted on the University of Michigan campus. Last, we describe the data needs to make such estimates accurate.

## Introduction

Timely and accurate estimates of influenza virus infection incidence rates in a population are difficult to obtain because most infectious episodes are unaccounted for, while influenza-like illness can have a variety of etiologies other than influenza virus infection. Many countries use sentinel surveillance systems to ascertain rates of medical consultations associated with influenza-like illness [1], [2], and these data typically track the influenza epidemic curve although they cannot provide information about absolute infection rates without additional data on sensitivity and specificity over time. Serological surveillance can permit robust estimates of incidence rates in a population, although it can be costly and has rarely been employed in the US. To date the literature only contains one serological study of influenza infection in the US during the 2009 H1N1 pandemic [3]. Under a number of assumptions, sentinel surveillance data can be combined with virologic surveillance to estimate infection rates using a multiplier-type approach [4].

Here we propose an alternative approach to estimating age-specific infection incidence rates in a population based on syndromic surveillance data. The idea is that different infections generate different distributions of symptom profiles for symptomatic individuals, and data on reported symptom profiles can be “deconvolved” to estimate the underlying incidence of various infections. For example, among children with acute respiratory illness (ARI), fever is predictive of influenza virus infection (hereafter referred to as influenza) as the etiology [5]. The proportion of febrile cases among individuals with ARI during a time period when influenza incidence is high is expected to be greater compared to the time period when influenza incidence is low. Data on symptomatic and febrile cases in age-stratified random population samples combined with an estimate of the proportion of influenza cases who develop fever can therefore potentially be used to infer influenza incidence during various time periods. More generally, using data on symptom profile distributions combined with symptom data collected through surveillance, one can estimate the symptomatic incidence attributable to each infection; the latter estimate is obtained using the Expectation-Maximization algorithm [6], [7]. Similar considerations, though involving different estimation methodology for symptoms and causes of death appeared in [8].

In this paper we present the deconvolution (estimation) process in detail and describe the data needs for making such estimates accurate. We test the effect of deconvolution on simulated incidence and symptom profile curves. Additionally, we apply it to syndromic data collected during the declining period of an influenza outbreak on the University of Michigan campus.

## Methods

### Ethics Statement

The study from which symptom profile data were obtained was approved by the Institutional Review Board at the University of Michigan (IRB # HUM00008566) under the “No more than minimal risk” classification (http://clinicaltrials.gov/ct2/show/NCT00490633).

### 1. Multinomial model

Suppose there are 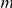 different pathogens causing infection in a population. Throughout this paper by “population” we denote a group of people assumed to be homogeneous in the sense than the distribution (probability) of symptoms associated with each of the various circulating pathogens does not change in time for the infected people in this population group. Correspondingly the estimation method for disease incidence is restricted to such a population for which symptom data is gathered.

Let the (unknown) symptomatic incidences of those infections in the population on week 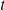 be 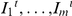 (we assume that no co-infections occur). Let 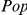
be the total population size, and let 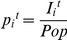
be the (time dependent) probability that a randomly selected person becomes symptomatic with infection 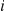 on week 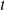.

Let 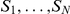 be the set of possible symptom profiles observed in patients. For instance if there are 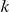 possible symptoms then one can take 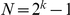
and the profiles are just the non-empty subsets of 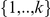. Use of this large number of profiles may lead to small counts of the numbers of people experiencing each symptom profile, so more parsimonious sets of symptom profiles may be advantageous.

For each infection 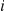 let 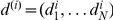
be the probabilities of particular symptom profiles for a person with that infection. For identifiability of different infections we assume that the matrix 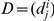
has rank 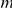, which in particular implies that 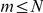 and that there is no infection for which the distribution of symptom profiles can be expressed as a linear combination of the other symptom profile distributions. Moreover we assume that for each 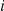,
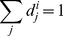
(*)Thus we are estimating symptomatic incidence, namely infections for which one of the chosen symptom profiles is present. Additional data on the proportion of individuals with each infection who are “asymptomatic” (do not exhibit any of the specified profiles) can allow one to estimate full incidence (symptomatic and asymptomatic incidence of the corresponding infection). For influenza, various estimates of the asymptomatic fraction exist in the literature [9], [10], [11]. A separate study tailored for the specific population and the circulating influenza strain should render a more accurate estimate of the asymptomatic fraction. Note that under this approach, estimation of the full incidence for the etiology of interest does not require data on the asymptomatic proportion for the other etiologies.

Suppose we have a weekly report from 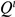
individuals on week 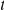 on their symptoms during the preceding week. The weekly data can be reduced to a vector 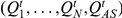 where 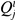 is the number of persons with symptom profile 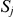 on week 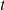, 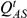 is the number of asymptomatic persons and
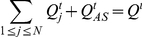
For each week 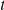 let 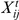 be the (unobserved) number of people among the 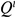 responders with infection 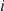 reporting symptom profile 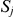
on week 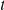 (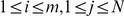). Thus
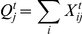
Moreover let 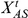 be the number of asymptomatic persons. For a randomly chosen person among the 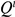 responders, the probability that he/she has infection 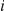 and reports symptom profile 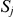 (falls into the category 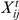) is 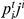. The probability that he/she is asymptomatic is 
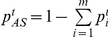
. Therefore the distribution of the (observed) symptom counts 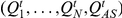 is multinomial with size 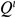 and the parameters
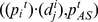
(1)where 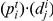 is a vector by matrix multiplication.

### 2. Symptom profile distributions

Equation (1) shows that the syndromic data alone cannot identify the number of symptomatic individuals with each infection. Therefore additional data on symptom profiles for various infections are needed for the inference process. More precisely, we assume that for each infection 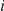 we have data on symptoms from 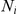 symptomatic individuals with infection 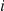. Let the observed counts of symptom profiles for those individuals be
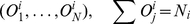
(**)Here the distribution of 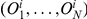 is multinomial of size 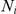 and parameters 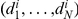. In this section we describe how such counts can be obtained from data; in the next section we describe the inference process using the symptom surveillance data and the symptom profile distribution data from equation (**).

Information about the distribution of symptoms for influenza can be obtained during the course of an epidemic, or from previous studies. We used the distribution of reported symptoms of individuals with influenza confirmed by real-time polymerase chain reaction (RT-PCR) in a community-based study [12]. Household contacts were recruited after index cases living in their household presented for medical care with influenza-like symptoms. A contact was deemed infected if at least one RT-PCR test was positive out of the 3 tests conducted during a 7-day follow-up period. A contact was deemed having fever if the maximal recorded tympanic temperature was 37.8°C or above. 118 influenza positive contacts in [12] had a presence of at least one of the following four signs or symptoms: fever, cough, runny nose, sore throat. We have examined the following two choices of symptom profiles for the inference process and estimated their distributions using data for those 118 individuals:

*Choice 1:*

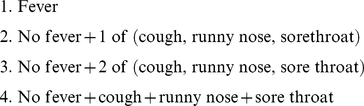
(2a)
*Choice 2:*

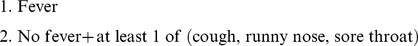
(2b)The distribution of symptom profiles reported by cases with ARI not associated with influenza may be quite specific to the location and the circulating respiratory viruses. One may be able to use symptom surveillance data itself during a period when one knows that the percent of flu among symptomatic individuals is very low to suggest that almost all symptoms are attributable to non-flu causes. Thus overall counts of symptom profiles reported by all symptomatic individuals during that period give an estimate of the non-flu symptom profile distribution. In this case that period should also be removed from subsequent inference of influenza incidence.

### 3. Inference process

#### 3.1 EM iterations

Estimation of the parameters 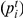
and 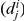 can be done with the aid of the Expectation Maximization (EM) algorithm [6], [7], [13] which iterates in the space of parameters 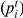
and 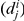 increasing the likelihood of observations 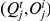 with each iteration. Specifically let 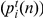
and 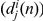
be the values of the parameters after 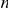 EM iterations. To understand the iterative process, denote for any parameters 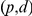 the expectation of the variable 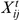 conditional on the observations 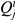 [13]:
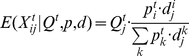
(3)The parameter values after the next EM iteration are given by
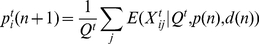
(4)and
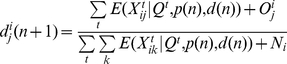
(5)


#### 3.2 Inference Method 1

Several inference methods for the model's parameters are possible, and their robustness for a given data set can be tested by the SEM algorithm [14], as well as by bootstrapping. Similarly our inference process involves a choice of certain symptom profiles for symptomatic individuals – e.g. the one given by equation (2a) or (2b). Assessing the robustness of each estimation method should also aid in the model selection for the inference process.

Method 1 essentially assumes that the distribution of symptoms is known. This deconvolution method based on equations (3) and (4) was introduced in the optics literature [15], [16] and subsequently used in the epidemiological literature [13], [17], [18]:

Fix the initial estimate of the symptom profile distribution
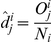
Using this estimate, iterate in the parameters 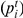 using equation (4) (keeping the parameters 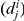 constant). Such iterates will converge to the unique maximum likelihood estimate 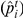 for the incidence parameters conditional on the parameters 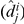 (see section S1) regardless of the initial choice of non-zero initial conditions.

#### 3.3 Inference Method 2

Other inference methods involve iterations in all of the model's parameters 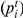 and 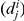
- the latter is generally known as “blind deconvolution” in the optics literature [19]. The advantage of those methods over Method 1 is that they allow to update the symptom profile distributions from the initial, data-derived estimate 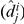 by increasing the likelihood of all observations 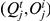. However given the lack of identifiability of the parameters using observations 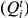 alone as specified by equation (1), iterations in the symptom profile distribution parameters might move them further away from their true value and worsen the incidence curve estimates if the counts 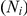 are too small.

Several inference methods involving EM iterations in all the parameters are possible, such as starting from an estimate in Method 1 and using both equations (4) and (5) for subsequent iterations. We have found that the classical EM scheme is fairly robust for sufficiently large data sets:

Iterate all the parameters simultaneously using both equations (4) and (5) in each step until convergence. An initial condition used in this paper corresponded to expected weekly incidence 1 for each infection in the survey sample (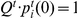).

### 4. Testing the deconvolution process by numerical simulations

To test the deconvolution process we generated synthetic weekly incidence curves both for influenza and non-influenza symptomatic cases over a 22-week period. The influenza incidence curve corresponds to an epidemic with basic reproductive number 1.35 and the serial interval distribution with mean 2.6 days [20] truncated at 7 days in a homogeneous population of 3,000,000.

We assumed that each week the number of individuals filling out the symptom survey is random, Poisson distributed with mean 5000. For our simulations, we used both choices of the symptom profiles described by equations (2a) and (2b), with their distribution for symptomatic flu cases estimated from the data in [12]. Similarly, for illustration purposes we have generated the non-flu symptom profile distribution for our simulations using the data from [21] (see section 5 of the methods).

We used weekly synthetic incidence and distribution of symptom profiles for flu and non-flu cases as described above to perform the following independent 3-step simulations:

1. Generate the (weekly) symptom count curves using the given incidence curves, symptom profile distributions and the weekly number of survey respondents.
2. Assume that the estimate of the symptom profile distribution 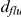 is obtained from data on 500 symptomatic flu cases. Re-estimate the influenza symptom profile distribution by multinomial binning of size 500 with the initial distribution 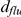. For non-flu symptoms, use the simulated symptom data from the first 3 weeks and the last 3 weeks (weeks 20–22) of the epidemic for an estimate the non-flu symptom profile distribution. During that period there are 2055 expected symptomatic cases given the incidence curves used in simulations and 99.6% of them are non-flu cases.
3. Using the symptom data in step 1 for weeks 4–19 and the re-estimates of the symptom profile distributions from step 2, apply the deconvolution scheme from the corresponding method; the output of the deconvolution process is an estimate of incidence between weeks 4–19.

We wish to point out that the accuracy of the deconvolution process depends not just on the number of individuals of survey but also on the level of circulation of influenza as well as other symptom causing pathogens in the community. Generally, accuracy would be higher if the counts for symptom profiles specific to influenza (primarily fever) in the survey are significantly larger than the magnitude of the noise in the corresponding counts for non-influenza symptomatic cases in the survey. In section S3 we perform various sensitivity analyses for the accuracy of the deconvolution process (Figures S1, S2 and S4).

### 5. University of Michigan outbreak

We have used symptom surveillance data from a randomized controlled trial of non-pharmaceutical interventions for preventing transmission of influenza collected in February–March 2008 on the University of Michigan campus [21]. With 1,000 individuals initially recruited, the weekly number of survey respondents ranged from 830 to 902. The eight weeks in the symptom surveillance data represent a period after an apparent peak of a seasonal influenza outbreak on the wider campus, as can be seem from data on influenza positive tests and ILI consultations on the campus medical facilities. The latter data suggest that flu circulation during the last two of the eight weeks was particularly low (figure S6 in section S5); surveillance data for those last two weeks in the survey was used to assess the non-flu symptom profile distribution. This symptom profile data from the last two weeks, available for 642 symptomatic individuals was combined with symptom data for the 118 RT-PCR positive household contacts of flu cases, as specified in section 2 of the methods to assess the influenza outbreak during weeks 1–6 in the survey period.

Weekly symptom profile counts attributable to influenza cases in the survey were estimated to be quite low, of the same magnitude as the noise (departure from expected values) in the symptom profile counts attributable to non-flu cases. As result, weekly estimates of flu incidence for such a small sample size in addition to having wide confidence bounds are also generally upwardly biased because they cannot go below 0. Consequently we have combined all the surveillance data and were only able to estimate the cumulative flu attack rate during the surveillance period, which the bootstrap simulations have shown to be unbiased.

## Results

### 1. Symptom profile distributions

**Figure 1:**
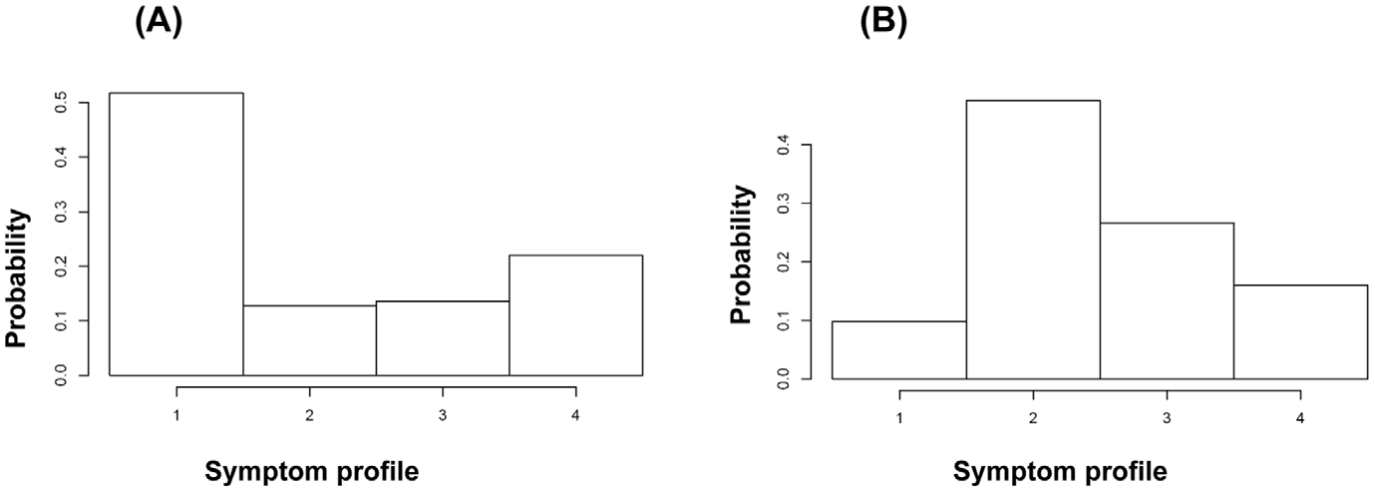
Distribution of symptom profiles for flu (A) and non-flu (B) symptomatic cases, inferred from data in [**12**] and [**21**]. Profile description is given in equation (2a).

Figure 1A plots the distribution of symptom profiles (as defined in equation (2a) in the Methods) for flu cases taken from [12], as described in section 3.1 of the methods. For our simulations we use the symptom profile distribution for non-flu cases obtained from the data in [21] (Figure 1B) – see also figure S5 in section S4.

Figure 1 suggests that fever is much more common for flu vs. non-flu cases, and one non-fever symptom only (cough, runny nose, or sore throat) is much more common for non-flu vs. flu cases.

### 2. Synthetic incidence and symptom data deconvolution

#### 2.1 Synthetic incidence curves

**Figure 2:**
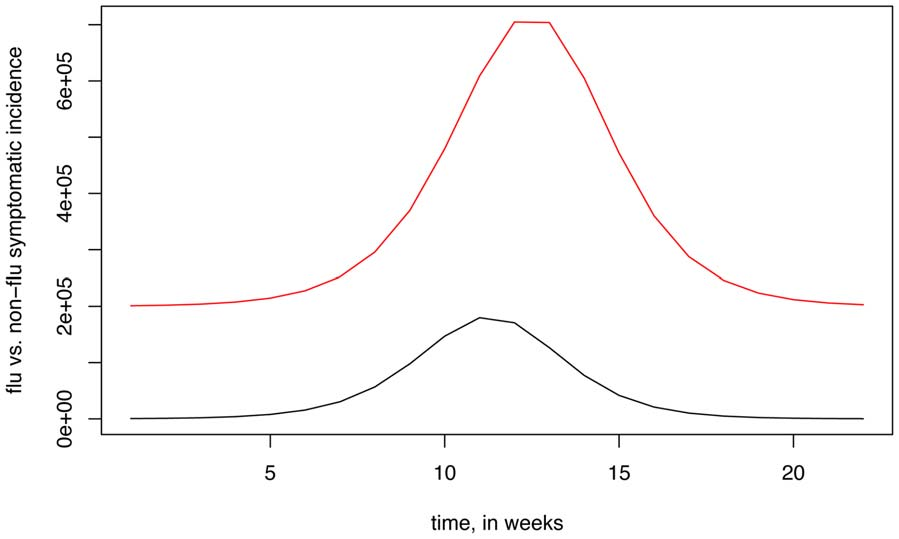
Synthetic weekly symptomatic incidence curves (as described in section 4 of the Methods) used to test the robustness of the deconvolution process: flu (black), non-flu (red).

We have generated synthetic incidence curves for flu and non-flu symptomatic cases as described in the Methods; those curves are plotted in Figure 2.

#### 2.2 Symptom profiles (2a)

**Figure 3:**
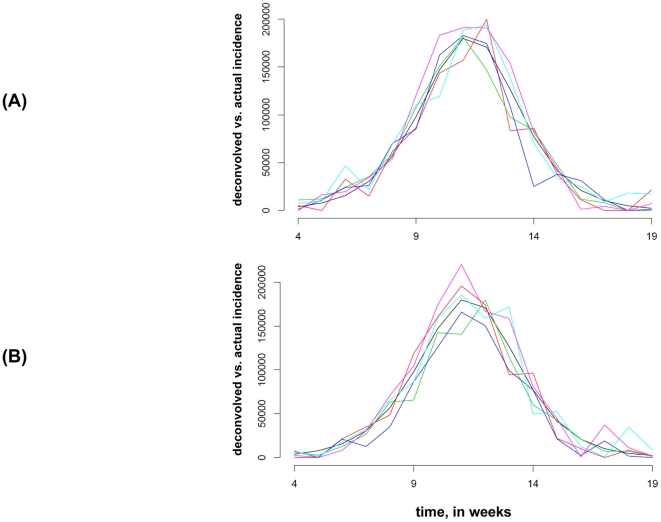
Two samples of 5 deconvolved influenza symptomatic incidence curves (as described in section 4 of the Methods) against the original one (black). (A) Method 1 deconvolution. (B) Method 2 deconvolution.

We have performed 600 3-step simulations as specified in section 4 of the methods, both for Method 1 and Method 2. Figure 3 plots two samples of 5 deconvolved influenza symptomatic incidence curves against the original one (black) between weeks 4–19. One sample is for Method 1 and another is for Method 2.

The cumulative number of symptomatic influenza cases between weeks 4 and 19 was 993,693. For Method 1, for the sample of 600 deconvolved symptomatic influenza incidence curves, their cumulative incidences have mean 978,266, with 95% of them falling between 762,556 and 1,197,579. For Method 2, the mean is 1,004,402, with the 95% range between 823,519 and1,185,116. We see that Method 2 gives a somewhat sharper estimate than Method 1 in this scenario.

#### 2.3 Symptom profiles (2b)

**Figure 4:**
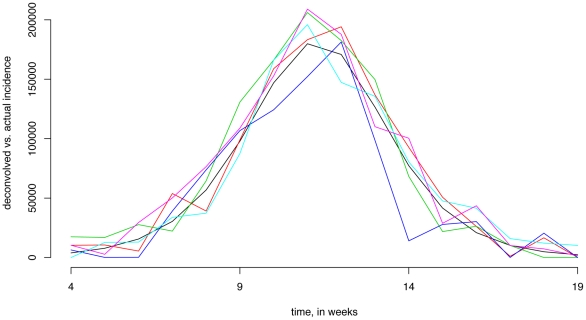
A sample of 5 deconvolved influenza symptomatic incidence curves (as described in section 4 of the Methods) against the original one (black) for symptom profiles (2b), deconvolution method 2.

Figure 4 plots a sample of 5 deconvolved influenza symptomatic incidence curves against the original one (black) between weeks 4–19, where symptom profiles (2b) and deconvolution Method 2 were used.

The cumulative number of symptomatic influenza cases between weeks 4 and 19 was 993,693. For the sample of 600 deconvolved symptomatic influenza incidence curves, their cumulative incidences have mean 1,011,040, with 95% of them falling between 793,340 and 1,209,297. We see that the estimates are somewhat better using symptom profiles (2a) than (2b) in this scenario.

### 3. University of Michigan outbreak

**Figure 5:**
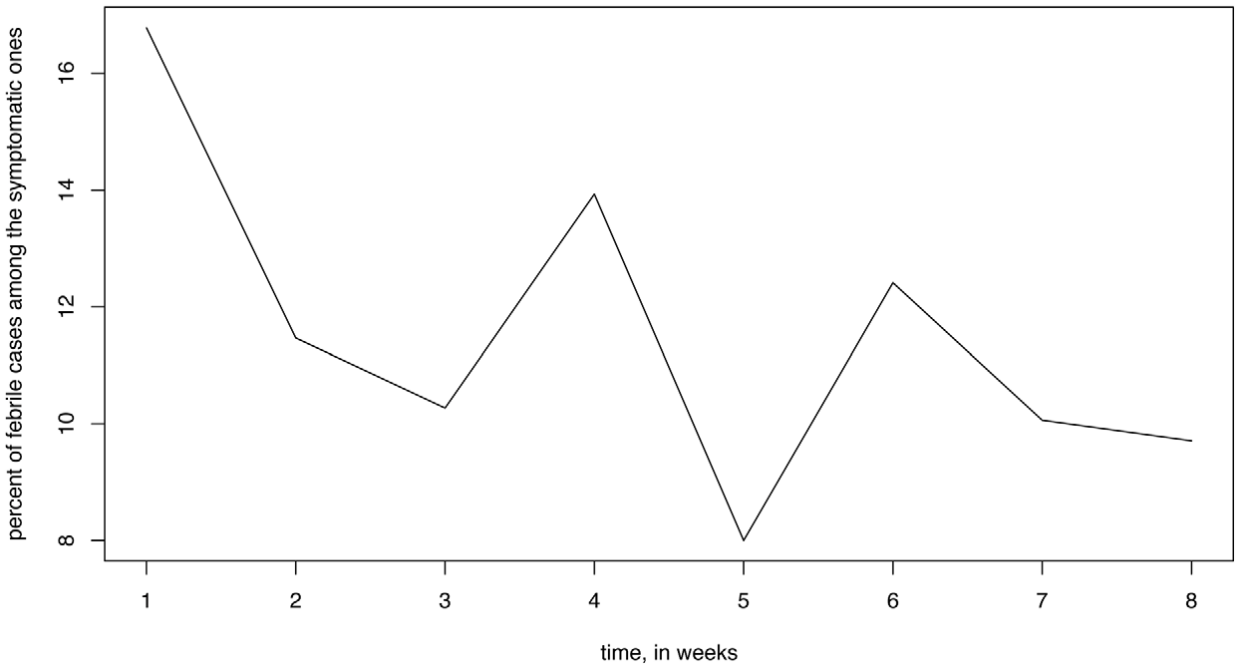
Weekly percent of cases with fever among the symptomatic cases in the survey from [**21**].

Figure 5 plots the weekly percentage of cases with fever among the symptomatic cases during weeks 1–8 in the survey data from [21]. This percentage declined towards the end of the survey period, reflecting the decline in the flu outbreak. The latter decline is statistically significant: for example during the first 3 weeks, 221/1555 (14.21%) of symptomatic individuals in the survey had fever; during the next 3 weeks, 108/1004 (10.76%) of symptomatic individuals had fever (OR 1.37, p-value 0.011 for the Fisher exact test).

The cumulative symptomatic attack rate of influenza during the first 6 weeks was estimated to be 15.3%; however the 95% confidence bounds were wide (2.2%,28.6%), suggesting that a larger survey sample size is needed for an accurate estimate.

## Discussion

Timely estimates of the progression of an influenza epidemic are difficult to obtain. Currently available surveillance methods render a limited assessment of the epidemic's growth patterns while serological surveillance is not commonly employed. Here we propose an alternative method to estimate incidence based on syndromic surveillance from population samples on regular times intervals. Such surveillance (e.g. [22]), combined with estimates of the distribution of symptom profiles for symptomatic influenza cases may, in principle, render an accurate estimate of the influenza incidence curve via the deconvolution process. We have proposed a collection of symptom profiles to be used in the deconvolution process, suggested how the corresponding symptom profile distributions can be estimated from data and tested the robustness of our method by numerical simulations. We wish to point out that while we restricted our methodology to influenza-like symptoms, it could in principle be adapted to estimation of incidence of other types of diseases (e.g. enteric infections), particularly if the infection of interest has a profile of symptoms which largely sets it apart from other related infections (similarly to the presence of fever, which is much more common for flu than for other respiratory infections).

The key potential limitation of our method is the ability to accurately estimate the distribution of symptom profiles for influenza and non-influenza cases. Estimate of the flu incidence is particularly sensitive to an estimate of the non-flu symptom profile distribution because there are many more symptomatic non-flu cases compared to the number of symptomatic flu cases in a survey, so a misattribution of a certain percentage of non-flu cases to flu is magnified relative to the flu data. Since the distribution of symptom profiles for non-flu cases may be specific to the given population, we propose to consider a time period in the surveillance data when very little influenza circulation is known to have taken place and use the symptom surveillance data for that period for an estimate of the symptom profile distribution for non-influenza cases. In this way, large sample size for the surveillance data would also ensure a more accurate estimate of the non-flu symptom profile distribution. Additionally, larger samples increase the size of the symptomatic counts attributable to influenza both in absolute terms and also relative to the noise in such counts attributable to non-flu cases, further improving the accuracy of the deconvolution process.

An additional potential issue with the symptom profile distribution for non-flu symptomatic cases is that it might change in time. The latter might occur due to an outbreak of a particular respiratory agent, such as human rhinovirus, coronavirus, or respiratory syncytial virus. The symptom profile distribution of these infections might be different from the overall distribution for symptomatic non-flu cases. One way to deal with this is to include this agent into the list of infections whose incidence is estimated through the deconvolution process. Alternatively, one may stick with flu and symptomatic non-flu cases as the two infectious profiles and use the excess fever approach (symptom profiles given by equation (2b)). The latter might still be robust because fever is much more common for flu than for non-flu cases and excess fever attributable to flu when flu circulation is sufficiently high should be larger than excess fever attributable to the potential difference in the probability of fever given non-flu symptoms during different time periods. This issue is examined through simulations in section S3, where a large non-flu outbreak with “atypical” symptoms is added as an unobserved component (Figure S3).

For the influenza symptom profile distribution, the most accurate estimates should be obtained using data for each specific (evolving) influenza season. Here for illustration purposes we have used data from [12] on RT-PCR positive household contacts recruited when a household index influenza case sought medical care. It is known that the accuracy of the PCR test is correlated with symptom presentation [23]. While three RT-PCR tests were administered on each household contact in [12], it is possible that some infected household contacts have tested negative, and this group is correlated with a weaker presentation of symptoms. Due to a relatively small sample size in [12] we did not attempt to derive age-stratified estimates of the symptom profile distribution. Some difference in symptom profile distribution for seasonal influenza A and B cases is possible (see section S2), though no statistically significant difference could be detected for the small sample of cases where sub-typing was performed. We believe that larger studies involving serology may render more accurate, age-stratified assessment of the syndrome distribution for seasonal influenza.

We have employed the above method for the data from a seasonal influenza outbreak on the University of Michigan campus. Those estimates have several potential limitations. The survey was not initially designed for our estimation method, with its size being too small for accurate estimates of influenza incidence. Data on symptom profiles for influenza used in the deconvolution process for the University of Michigan campus is obtained from a different population in [12]. Our assumption, based on the campus medical facilities data, that the influenza outbreak has waned towards the end of the study period may not be representative of the whole University of Michigan campus. Therefore our estimates for the University of Michigan outbreak are mostly given for illustrative purposes. A careful study design should be used to avoid some of those issues. Such design should perhaps involve the recruitment of a large number of individuals (larger than what is needed for a weekly survey) with a commitment from them to complete a certain number of surveys when prompted during the study period. The latter should decrease the correlation between the weekly symptom reports and increase the percent of weekly recruits who fill out a report, taking away from the recruitment bias when participation might be correlated with symptom presentation. Finally, serologic data if available could validate the syndrome-based estimation of infection attack rates.

## Supporting Information

Section S1 **Convexity of the log likelihood function.**
(DOC)

Section S2 **Influenza A and B symptom profiles.**
(DOC)

Section S3 **Numerical simulations for the deconvolution process.**
(DOC)

Section S4 **Symptom profile distribution for PCR-negative, symptomatic household contacts.**
(DOC)

Section S5 **Influenza incidence proxy on the University of Michigan campus.**
(DOC)

Figure S1 **Two samples of 5 deconvolved influenza symptomatic incidence curves (as described in section S3) against the original one (black).** (A) Method 1 deconvolution. (B) Method 2 deconvolution.
(TIF)

Figure S2 **A sample of 5 deconvolved influenza symptomatic incidence curves (as described in section S3) against the original one (black).** Symptom profiles (2b), Method 2.
(TIF)

Figure S3 **Adding an “unobserved” non-flu outbreak with atypical symptoms (as described in section S3).** Symptomatic influenza incidence (black), “regular” non-flu incidence (red) and “outbreak” non-flu incidence (dashed red).
(TIF)

Figure S4 **The effect of an “unobserved” non-flu outbreak with atypical symptoms (as described in section S3) on the deconvolution process.** A sample of 5 deconvolved influenza symptomatic incidence curves against the original one (black). Symptom profiles (2b), Method 2. Flu and non-flu incidence curves given by Figure S3.
(TIF)

Figure S5 **Symptom profile distribution for PCR negative, symptomatic household contacts from** [**12**] **(A).** Non-flu symptom profile distribution from the main body of the text (B).
(TIF)

Figure S6 **Weekly incidence proxy on the University of Michigan campus, inferred from survey and virological testing data in** [**21**] **.**
(TIF)
