# Supplementary material for: Estimating Incidence Curves of Several Infections Using Symptom Surveillance Data
Source: PLoS One. 2011 Aug 24;6(8):e23380. doi: 10.1371/journal.pone.0023380 (PMC3160845; doi:10.1371/journal.pone.0023380)
Supplement: Section S1 — Convexity of the log likelihood function. (DOC) [file pone.0023380.s001.doc]

**Section S1: Convexity of the log likelihood function**

In this section we show that log likelihood of the observed symptom profile counts is a convex function of the incidence parameters (the matrix of its second derivatives is negative definite) if the distribution of symptoms matrix $(d_{j}^{i})$ has full rank (equaling the number $m$ of possible pathogens). This in particular implies that the EM iterations will converge to the unique maximum likelihood estimate (given the matrix $(d_{j}^{i})$) if the limit is in the interior.

Let $(p_{1},\ldots,p_{m+1})$ be the population proportions of symptomatic infections associated with the different pathogens on some week (including the asymptomatic proportion $p_{m+1}$) and let $(X_{1},\ldots,X_{N+1})$ be the symptom profile counts on that week (including no symptoms $X_{N+1}$). We extend the matrix $(d_{j}^{i})$ to be an $(N+1)\times(m+1)$ matrix having a bottom row and a rightmost column of zeroes except for the bottom right element, which is 1 – the new matrix, which we still call $(d_{j}^{i})$, is the distribution of possible outcomes given different states, and it still has full rank.

The multinomial log likelihood (minus the constant term of the logarithm of the multinomial coefficient) is

$$g(p_{1},\ldots,p_{m+1})=\sum_{j=1}^{N+1} X_{j}\cdot log(\sum_{i=1}^{m+1} p_{i}\cdot d_{j}^{i})$$

The matrix of its second derivatives with respect to the variables $(p_{i})$ is

$$Hess(g)=(\frac{\partial^{2}g}{\partial p_{i}\partial p_{k}})=(\sum_{j=1}^{N+1} \frac{-X_{j}d_{j}^{i}d_{j}^{k}}{(\sum_{l=1}^{N+1} p_{l}d_{j}^{l})^{2}})$$

One readily sees that$Hess(g)=-M\cdot M^{T}$, where $M$is the $(m+1)\times(N+1)$matrix with

$M_{ij}=\frac{\sqrt{X_{j}}\cdot d_{j}^{i}}{\sum_{l=1}^{N+1} p_{l}d_{j}^{l}}$.

This means that for any vector $v$, the second derivative of $g$in the direction of $v$ at point $p=(p_{1},\ldots,p_{m+1})$ (along a line $p+tv$) is

$$\frac{\partial^{2}g}{\partial v^{2}}=v^{T}\cdot Hess(g)\cdot v=-||M^{T}\cdot v||^{2}\leq0$$

and the latter is strictly negative if $v\neq0$ by the assumption that $(d_{j}^{i})$ has full rank (because the columns of $M$, which are the rows of $M^{T}$, are proportional to the columns of $(d_{j}^{i})$, hence $M^{T}$ has a zero kernel).
