# Supplementary material for: Estimating Incidence Curves of Several Infections Using Symptom Surveillance Data
Source: PLoS One. 2011 Aug 24;6(8):e23380. doi: 10.1371/journal.pone.0023380 (PMC3160845; doi:10.1371/journal.pone.0023380)
Supplement: Section S2 — Influenza A and B symptom profiles. (DOC) [file pone.0023380.s002.doc]

**Section S2: Influenza A and B symptom profiles**

Fifty two PCR positive household contacts from had their sample sub-typed. Among those, 33 had influenza A (H3N2 or H1N1) and 19 had influenza B. The frequency of symptom profiles for influenza A was (18,4,4,7); the frequency of symptom profiles for influenza B was (8,5,2,4). No statistically significant difference between those distributions was found (p-value 0.64 for the Fisher exact test). Larger studies are needed to estimate symptom profile distributions specific to a type/subtype of influenza.

1. Cowling BJ, Chan KH, Fang VJ, Cheng CK, Fung RO, et al. (2009) Facemasks and hand hygiene to prevent influenza transmission in households: a cluster randomized trial. Ann Intern Med 151: 437-446.
