# Supplementary material for: Estimating Incidence Curves of Several Infections Using Symptom Surveillance Data
Source: PLoS One. 2011 Aug 24;6(8):e23380. doi: 10.1371/journal.pone.0023380 (PMC3160845; doi:10.1371/journal.pone.0023380)
Supplement: Section S3 — Numerical simulations for the deconvolution process. (DOC) [file pone.0023380.s003.doc]

**Section S3: Numerical simulations for the deconvolution process**

In this section we perform additional simulations testing the robustness of the deconvolution method. These simulations are identical to those in section 4 of the Methods with the exception of step 2 where smaller samples for the symptomatic flu and non-flu cases are assumed to be available. More precisely, the following 3-step simulations are performed:

1(SI): Generate the (weekly) symptom count curves using the given incidence curves (Figure 2), symptom profile distributions (Figure 1) and the weekly number of survey takers.

2(SI): Assume that the estimate of the symptom profile distribution $d_{flu}$ is obtained from data on 200 symptomatic flu cases. Re-estimate the influenza symptom profile distribution by multinomial binning of size 200 with the initial distribution $d_{flu}$. For non-flu symptoms, use the simulated symptom data from the first 2 weeks and the last week (week 22) of the epidemic for an estimate the non-flu symptom profile distribution. During that period there are 1013 expected symptomatic cases given the incidence curves used in simulations and 99.7% of them are non-flu cases.

3(SI): Using the symptom data in step 1 for weeks 3-21 and the re-estimates of the symptom profile distributions from step 2, apply the deconvolution scheme from the corresponding method; the output of the deconvolution process is an estimate of incidence between weeks 3-21.

***Symptom profiles (2a)***

Figure S1 plots two samples of 5 deconvolved influenza symptomatic incidence curves against the original one (black) between weeks 3-21. One sample is for Method 1 and another is for Method 2.

The cumulative symptomatic influenza attack rate between weeks 3 and 21 was 997,191. For Method 1, for the sample of 600 deconvolved symptomatic influenza incidence curves, their cumulative incidences have mean 996,885, with the 95% range from 683,319 to 1,278,877. For Method 2, the mean is 1,091,561, and the 95% range is 851,303 to 1,344,421. We see that under the current scenario, which has a smaller sample size for symptom profile counts both for flu and non-flu cases compared to what was considered in the main body of the text, accuracy of the deconvolution process has decreased and Method 2 presents an upward bias in influenza incidence estimation.

***Symptom profiles (2b)***

Figure S2 plots a sample of 5 deconvolved influenza symptomatic incidence curves against the original one (black) between weeks 3-21, where symptom profiles (2b) and deconvolution Method 2 were used.

The cumulative symptomatic influenza attack rate between weeks 3 and 21 was 997,191. For the sample of 600 deconvolved symptomatic influenza incidence curves, their cumulative incidences have mean 1,074,571, and the 95% range is 869,751 to 1,303,111. We see that with a smaller sample for symptom data in this scenario, a similar upward bias exists in the estimate of the cumulative influenza attack rate using symptom profiles (2a) and (2b); however in the latter case the confidence bounds are tighter. Note that this is the opposite trend from that found in the main text, where symptom profile (2b) gave wider confidence bounds; this suggests that when data are sufficient, a more detailed approach (profile 2a) is warranted, but when data become scarce, a simpler approach (2b) gives better performance.

***Time-varying symptom profile distribution for non-flu cases***

This section studies the deconvolution process under time-varying distribution of symptom profiles for non-flu cases. The latter variability is modeled by introducing an outbreak of a respiratory agent for which 15% of symptomatic cases have fever. This outbreak occurs in addition to “regular” non-flu cases whose incidence curve is identical to the one used in all other simulations and for whom 9.8% of symptomatic cases have fever, as before. The symptomatic incidence curve for influenza cases is also identical to the one used in all other simulations, with 51.7% of symptomatic flu cases being febrile as before. Figure S3 plots the three incidence curves.

We assume no knowledge about the non-flu outbreak from Figure S3 in our inference process. Correspondingly, we perform 600 following 3-step simulations:

A(S): Generate the (weekly) fever + symptomatic non-fever count curves using the given incidence curves (Figure S3), probabilities of fever given symptoms described earlier in this section and the weekly number of survey takers.

B(S): Assume that the estimate of the symptom profile distribution $d_{flu}$ is obtained from data on 200 symptomatic flu cases. Re-estimate the influenza symptom profile distribution by multinomial binning of size 200 with the initial distribution $d_{flu}$. For non-flu symptoms, use the simulated symptom data from the last 3 weeks (weeks 20-22) of the epidemic (when flu and “outbreak” incidences are negligible) for an estimate of the non-flu symptom profile distribution. During that period there are 1039 expected symptomatic cases given the incidence curves used in simulations and 99.7% of them are non-flu cases.

C(S): Using the symptom data in step 1 for weeks 1-19 and the re-estimates of the symptom profile distributions from step 2, apply the deconvolution scheme using Method 2; the output of the deconvolution process is an estimate of incidence between weeks 1-19.

Figure S4 plots a sample of 5 deconvolved flu incidence curves against the original one (black) between weeks 1-19. The original influenza attack rate between weeks 1-19 was 996,997. For the sample of 600 deconvolved symptomatic influenza incidence curves, their cumulative incidences have mean 1,170,422, and the 95% range is 891,793 to 1,444,202. We see that having introduced an outbreak with a higher probability of fever than “regular” non-flu symptomatic cases (which formed the bulk of the symptom data during the last 3 weeks in the survey period), the upward bias in the estimate of influenza attack rate has increased compared to the previous section and the confidence bounds became wider.
