# Supplementary material for: Estimating Incidence Curves of Several Infections Using Symptom Surveillance Data
Source: PLoS One. 2011 Aug 24;6(8):e23380. doi: 10.1371/journal.pone.0023380 (PMC3160845; doi:10.1371/journal.pone.0023380)
Supplement: Section S4 — Symptom profile distribution for PCR-negative, symptomatic household contacts. (DOC) [file pone.0023380.s004.doc]

**Section S4: Symptom profile distribution for PCR-negative, symptomatic household contacts**

In our primary analysis we’ve used data on symptoms for PCR-positive household contacts from to estimate the symptom profile distribution for influenza, and data on symptoms during a period of apparently very low circulation of influenza on a University of Michigan campus from to estimate the symptom profile distribution for symptomatic non-flu cases. One may wonder how compatible are the symptom data in Hong Kong and on the University of Michigan campus. To that end we’ve examined symptom data for household contacts in the Hong Kong study who were negative for the three PCR tests administered during a 7-day follow-up period in their household. One should note that due to the imperfection of the PCR test, some of those individuals could have been false negative influenza cases. Nonetheless we compare the symptom profile distribution for those individuals with the non-flu symptom profile distribution estimated on the University of Michigan campus (Figure S5). We note that there is a good deal of similarity between those two distributions, particularly in the percentage of symptomatic cases who are febrile (symptom profile 1).

1. Cowling BJ, Chan KH, Fang VJ, Cheng CK, Fung RO, et al. (2009) Facemasks and hand hygiene to prevent influenza transmission in households: a cluster randomized trial. Ann Intern Med 151: 437-446.

2. Aiello AE, Monto, A.S. (2009) Reducing transmission of influenza by face masks and hand hygiene. Presentation to the IOM Committee on Respiratory Protection for Healthcare Workers in the Workplace Against Novel H1N1 Influenza A, August 12, 2009. Washington, DC.
