# Supplementary material for: Estimating Incidence Curves of Several Infections Using Symptom Surveillance Data
Source: PLoS One. 2011 Aug 24;6(8):e23380. doi: 10.1371/journal.pone.0023380 (PMC3160845; doi:10.1371/journal.pone.0023380)
Supplement: Section S5 — Influenza incidence proxy on the University of Michigan campus. (DOC) [file pone.0023380.s005.doc]

**Section S5: Influenza incidence proxy on the University of Michigan campus**

This section presents additional data on the influenza outbreak on the University of Michigan campus during the 8 weeks of the symptom surveillance period. Each week, a certain fraction of the survey participants reported influenza-like-illness (ILI), which was defined in as cough and at least one of the constitutional symptoms of chills, feverishness, body aches. A certain fraction of ILI cases in the survey have reported to campus health care facilities, and presence of influenza among those individuals was tested using RT-PCR. Figure S6 plots the weekly incidence proxy of influenza on campus obtained using these data, where the proxy is defined as the percent of survey takers who reported ILI times the percent of ILI cases who tested positive for influenza on the campus medical facilities.

We want to point out that this proxy has a number of limitations. Individuals who presented to campus medical facilities presumably had stronger symptoms than an average individual reporting ILI in the survey, and stronger symptom presentation could be correlated with the presence of influenza – thus influenza rates in ILI cases who presented to medical facilities are likely to be higher than influenza rates among the survey ILI cases. On the other hand false negative results are known to take place for the RT-PCR testing, potentially underestimating the latter rates. Finally, not all influenza infections are accompanied by ILI. Thus altogether the incidence proxy in Figure S6 is at best a multiplicative proxy of incidence. Nonetheless Figure S6 clearly shows that influenza incidence rates on campus were declining rapidly during the study period, which is correlated with the decline in the percent of febrile cases among the symptomatic individuals in the survey in Figure 5 in the main text.

1. Aiello AE, Monto, A.S. (2009) Reducing transmission of influenza by face masks and hand hygiene. Presentation to the IOM Committee on Respiratory Protection for Healthcare Workers in the Workplace Against Novel H1N1 Influenza A, August 12, 2009. Washington, DC.
