# Supplementary figures and images for: Estimating Incidence Curves of Several Infections Using Symptom Surveillance Data
Source: PLoS One. 2011 Aug 24;6(8):e23380. doi: 10.1371/journal.pone.0023380 (PMC3160845; doi:10.1371/journal.pone.0023380)

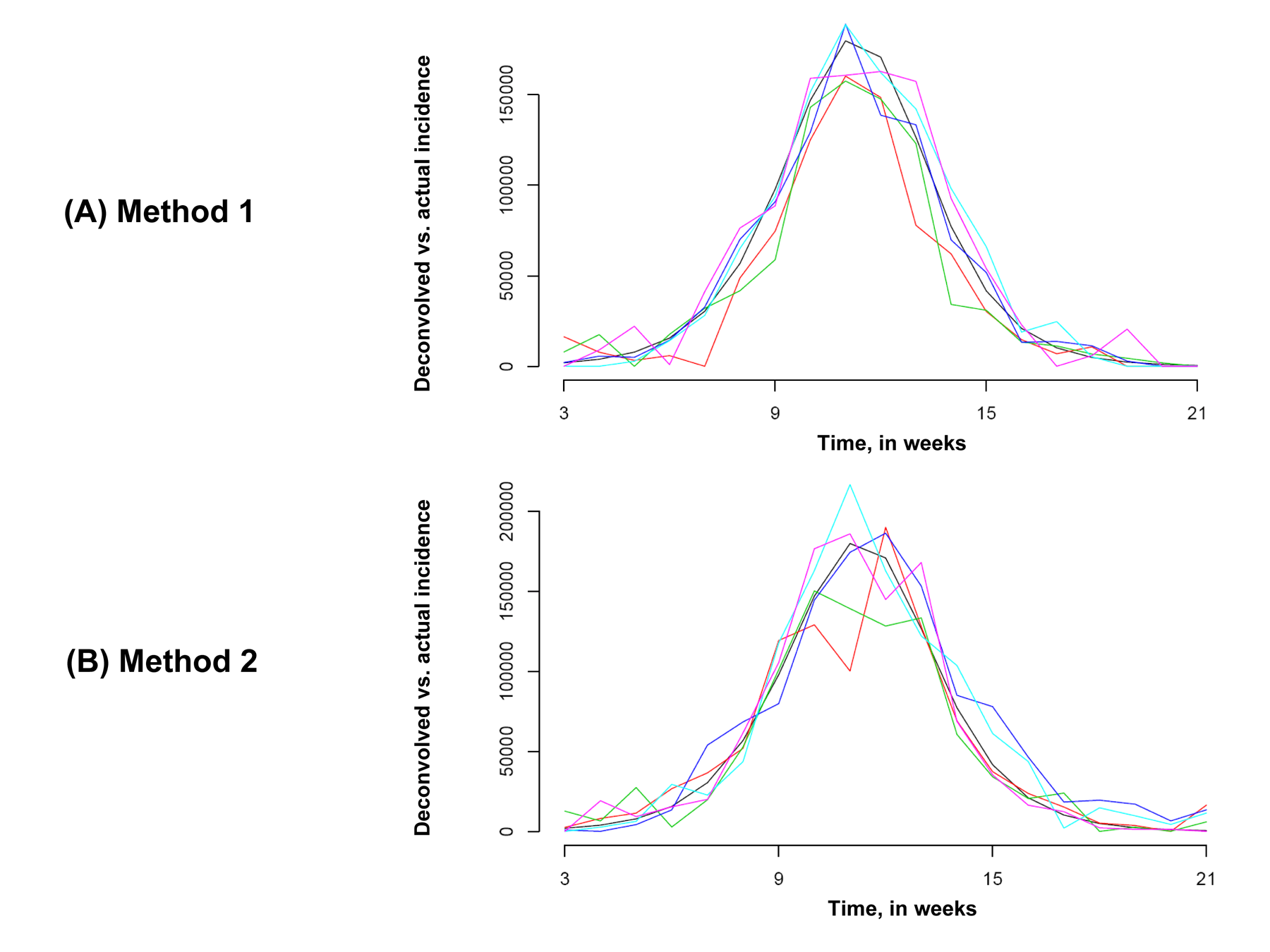

Supplement: Figure S1 — Two samples of 5 deconvolved influenza symptomatic incidence curves (as described in section S3) against the original one (black). (A) Method 1 deconvolution. (B) Method 2 deconvolution. (TIF) [file pone.0023380.s006.tif]

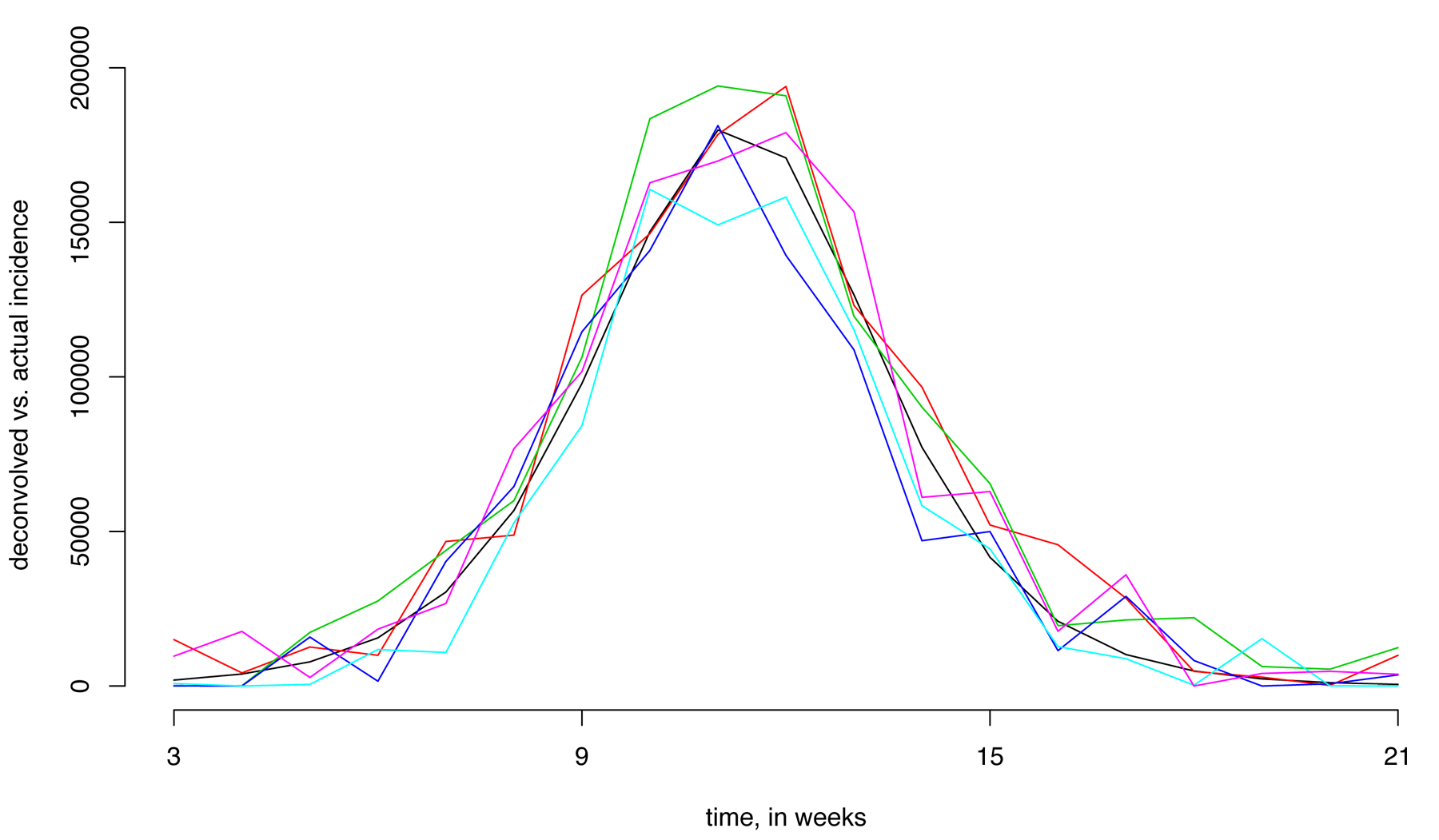

Supplement: Figure S2 — A sample of 5 deconvolved influenza symptomatic incidence curves (as described in section S3) against the original one (black). Symptom profiles (2b), Method 2. (TIF) [file pone.0023380.s007.tif]

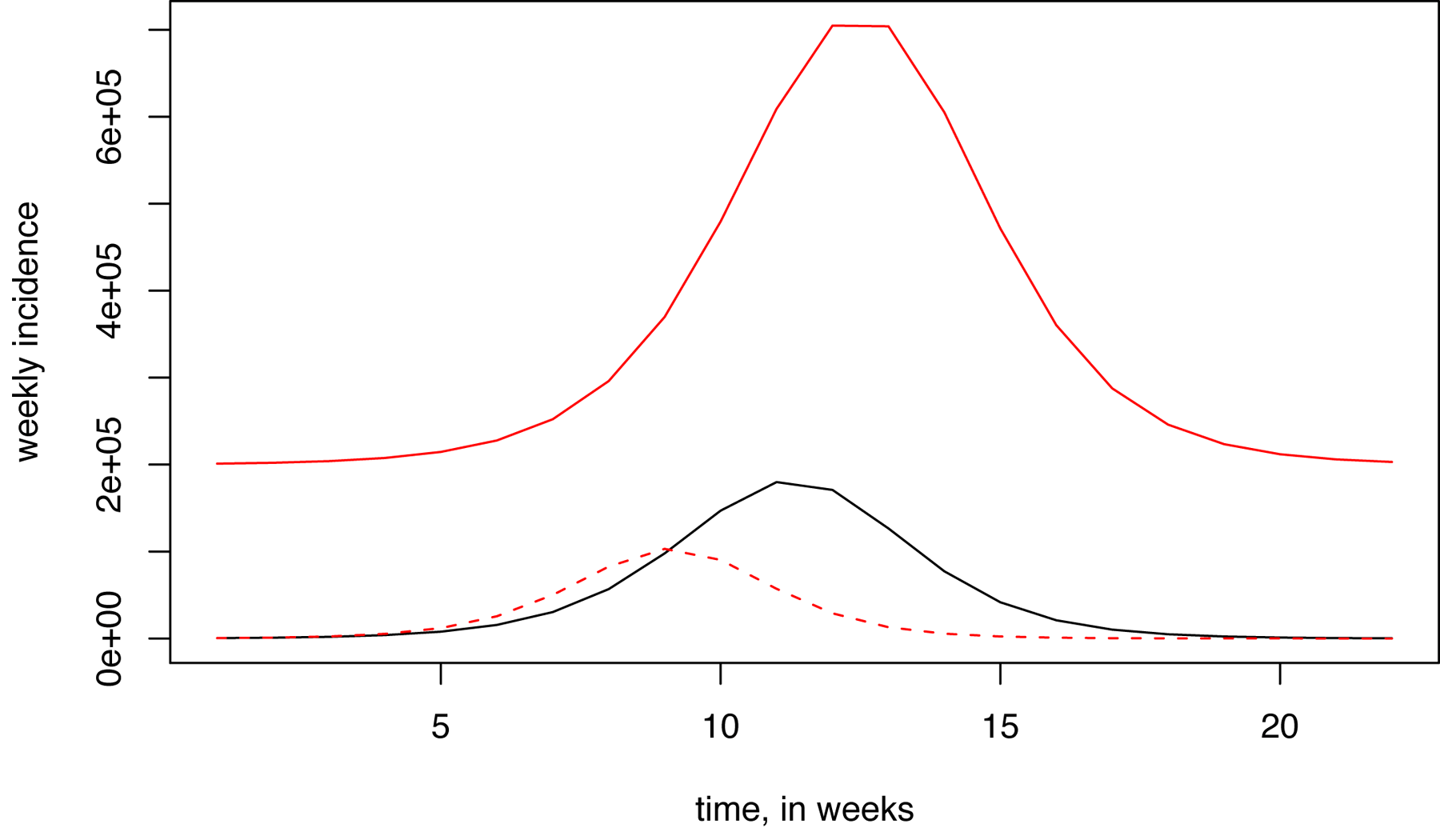

Supplement: Figure S3 — Adding an “unobserved” non-flu outbreak with atypical symptoms (as described in section S3). Symptomatic influenza incidence (black), “regular” non-flu incidence (red) and “outbreak” non-flu incidence (dashed red). (TIF) [file pone.0023380.s008.tif]

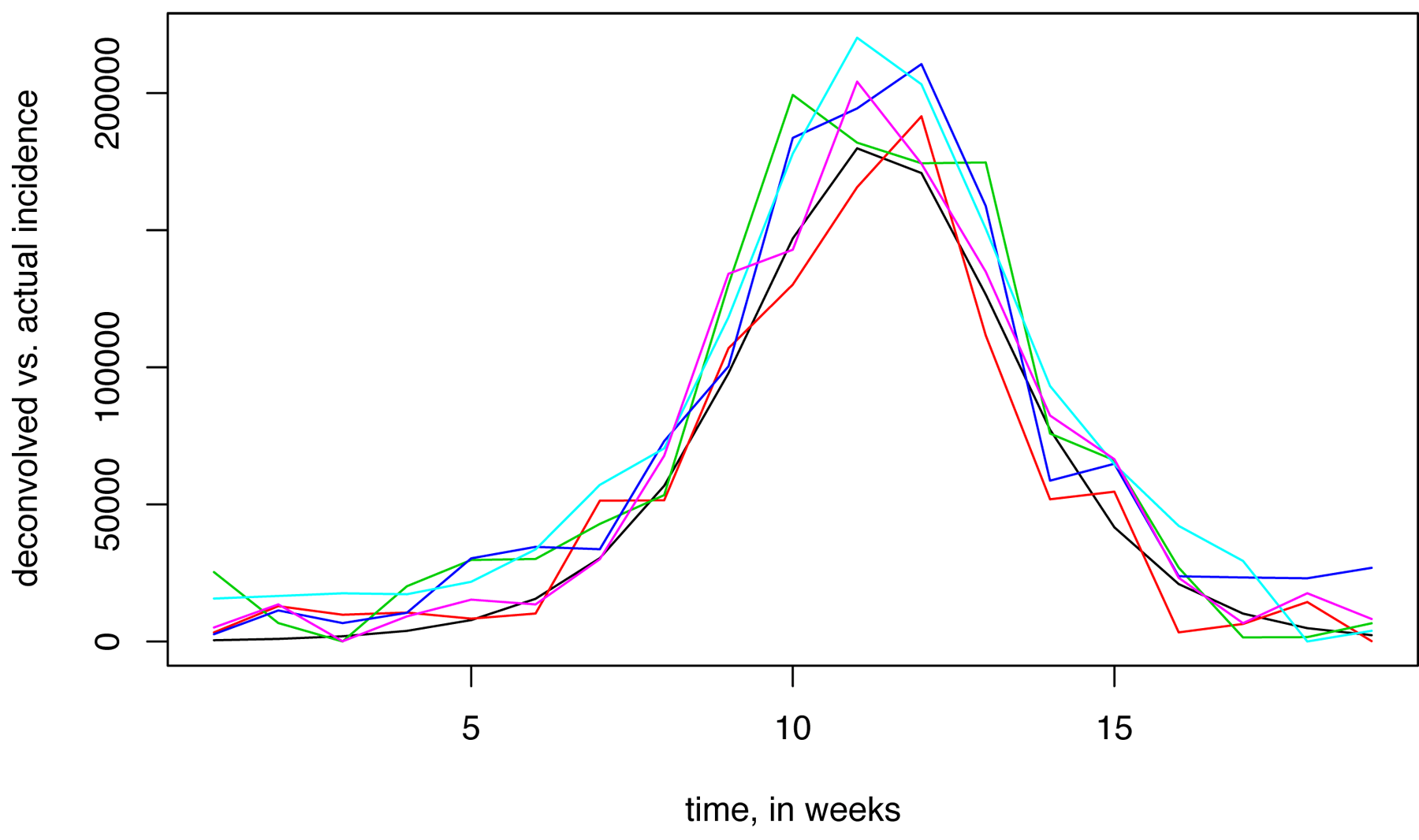

Supplement: Figure S4 — The effect of an “unobserved” non-flu outbreak with atypical symptoms (as described in section S3) on the deconvolution process. A sample of 5 deconvolved influenza symptomatic incidence curves against the original one (black). Symptom profiles (2b), Method 2. Flu and non-flu incidence curves given by Figure S3. (TIF) [file pone.0023380.s009.tif]

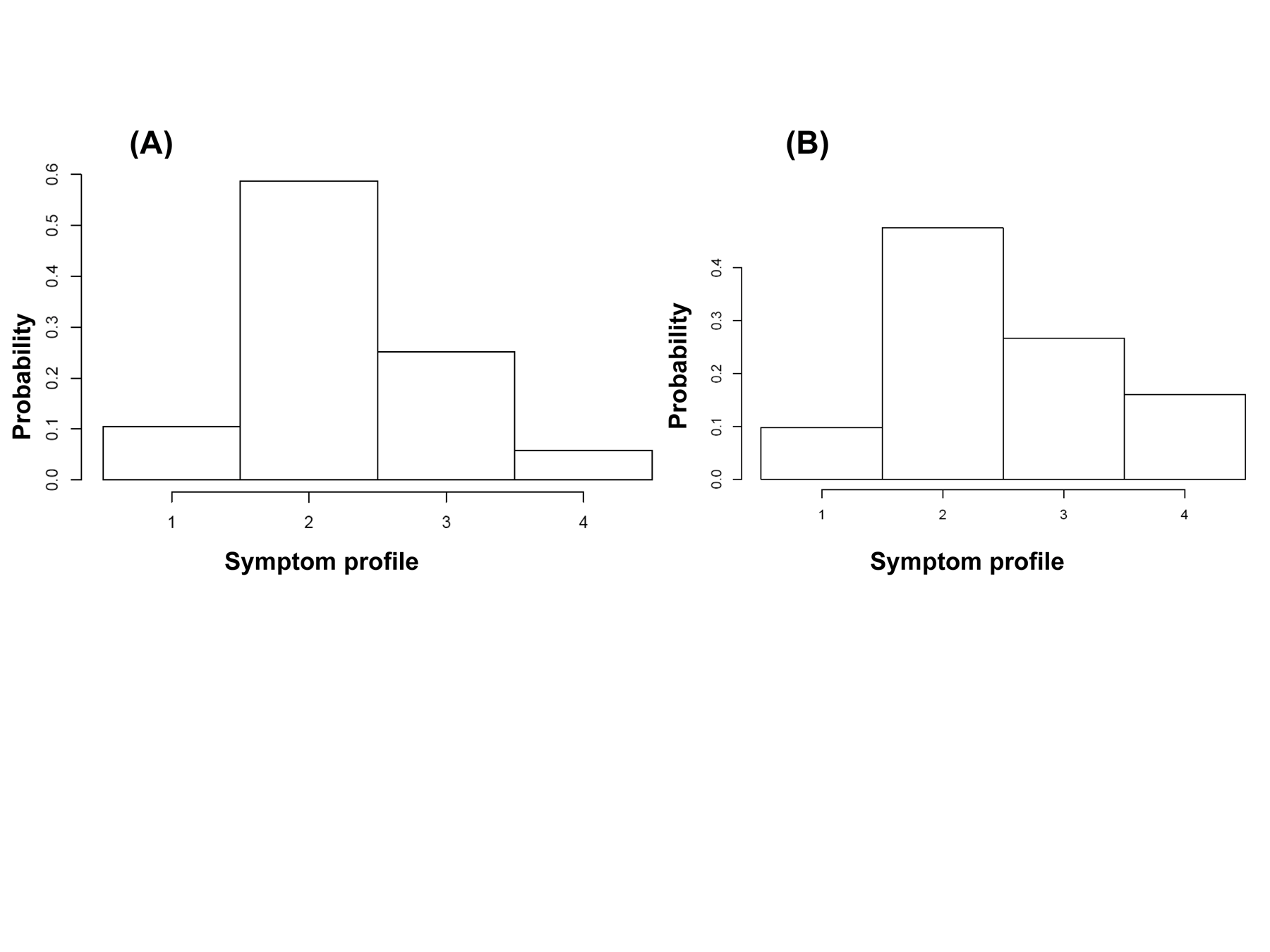

Supplement: Figure S5 — Symptom profile distribution for PCR negative, symptomatic household contacts from [12] (A). Non-flu symptom profile distribution from the main body of the text (B). (TIF) [file pone.0023380.s010.tif]

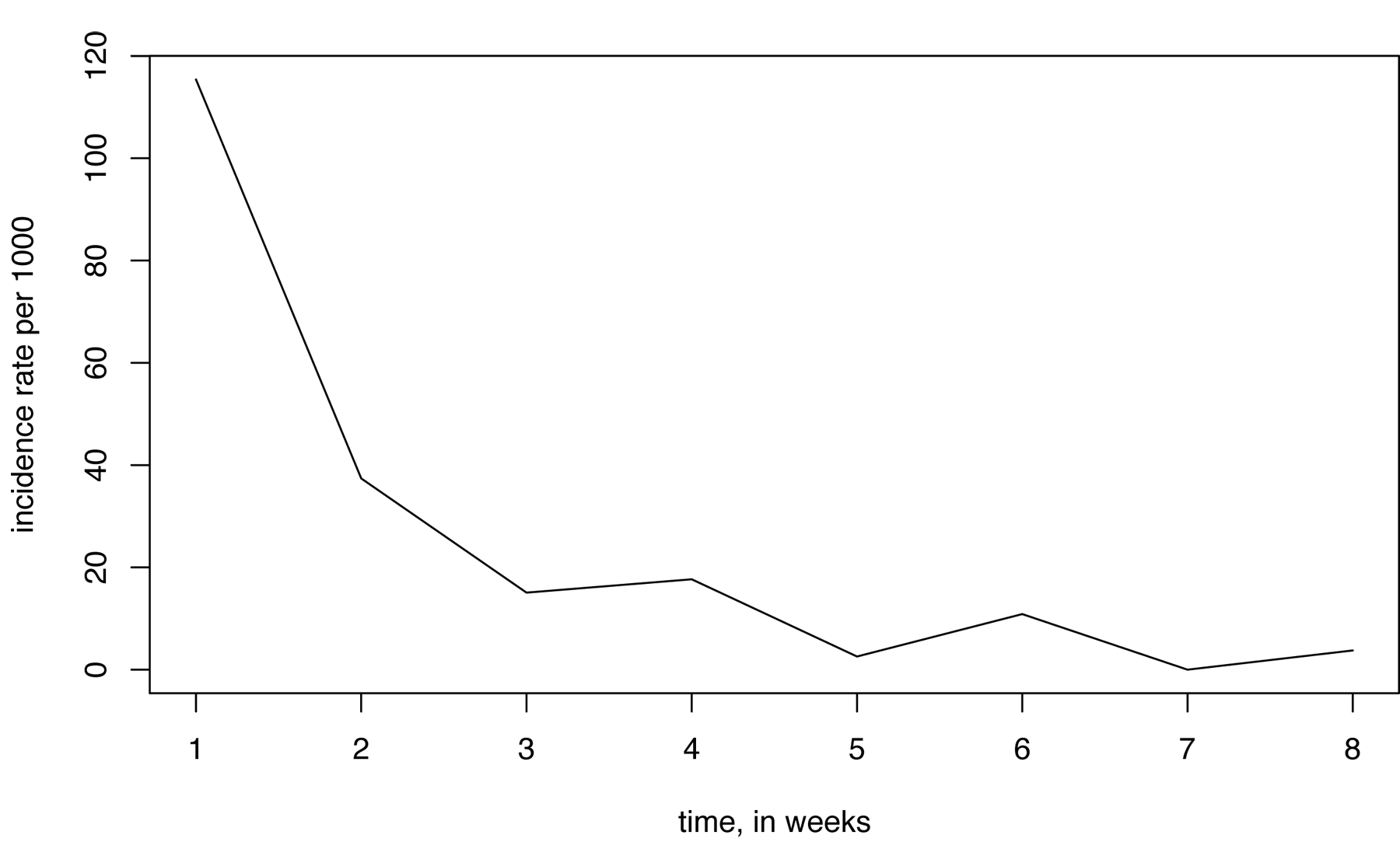

Supplement: Figure S6 — Weekly incidence proxy on the University of Michigan campus, inferred from survey and virological testing data in [21] . (TIF) [file pone.0023380.s011.tif]
